# The Optimal Radiation Dose to Induce Robust Systemic Anti-Tumor Immunity

**DOI:** 10.3390/ijms19113377

**Published:** 2018-10-29

**Authors:** Jan Poleszczuk, Heiko Enderling

**Affiliations:** 1 Nalecz Institute of Biocybernetics and Biomedical Engineering, Polish Academy of Sciences, Ks. Trojdena 4, 02-109 Warsaw, Poland; 2 Department of Integrated Mathematical Oncology, H. Lee Moffitt Cancer Center and Research Institute, 12902 Magnolia Drive, Tampa, FL 33612, USA; heiko.enderling@moffitt.org; 3 Department of Radiation Oncology, H. Lee Moffitt Cancer Center and Research Institute, 12902 Magnolia Drive, Tampa, FL 33612, USA

**Keywords:** abscopal effect, radiotherapy, immunotherapy, mathematical model

## Abstract

The synergy of radiation and the immune system is currently receiving significant attention in oncology as numerous studies have shown that cancer irradiation can induce strong anti-tumor immune responses. It remains unclear, however, what are the best radiation fractionation protocols to maximize the therapeutic benefits of this synergy. Here, we present a novel mathematical model that can be used to predict and dissect the complexity of the immune-mediated response at multiple tumor sites after applying focal irradiation and systemic immunotherapy. We successfully calibrate the proposed framework with published experimental data, in which two tumors were grown in mice at two spatially-separated sites from which only one was irradiated using various radiation fractionation protocols with and without concurrent systemic immunotherapy. The proposed model is calibrated to fit the temporal dynamics of tumor volume at both sites and can predict changes in immune infiltration in the non-irradiated tumors. The model was then used to investigate additional radiation fractionation protocols. Model simulations suggest that the optimal radiation doses per fraction to maximize anti-tumor immunity are between 10 and 13 Gy, at least for the experimental setting used for model calibration. This work provides the framework for evaluating radiation fractionation protocols for radiation-induced immune-mediated systemic anti-tumor responses.

## 1. Introduction

**Figure 1:**
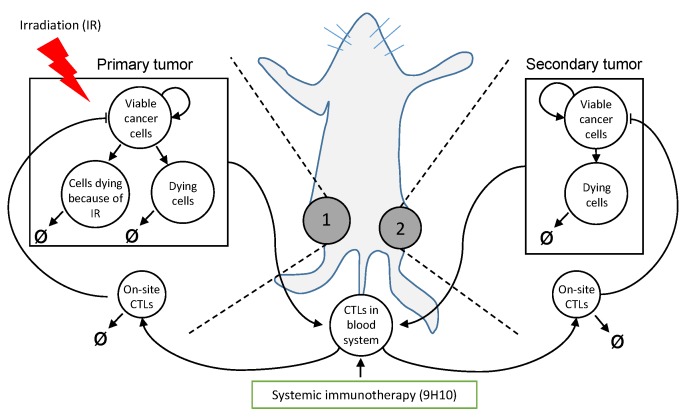
Schematic of the experimental setting and model compartments (circles). Arrows indicate transitions between compartments and T bars indicate killing of cancer cells by immune cells. The proposed model (Equations (3)–(10)) formalizes the relevant mechanisms underlying the in vivo experiment [11] in which carcinoma cells were injected subcutaneously into mice at two spatially-separated sites (left and right flank), and only one site (1, primary tumor) was later irradiated (IR). Some mice received also systemic immunotherapy (9H10), which augments activation of tumor-specific cytotoxic T lymphocytes (CTLs).

Radiation is a potent cancer treatment to sterilize abnormal or potentially abnormal tissues locally. About 50% of all cancer patients receive radiotherapy as part of their treatment, either as monotherapy or in combination with surgery, chemotherapy or, more recently, immunotherapy [1,2]. Despite decades of clinical success that have seen tremendous physical advances, some questions in radiobiology remain elusive including how to trigger the radiation-induced bystander [3,4,5] and the abscopal [6,7] effects reliably to further increase therapeutic efficacy. The abscopal effect, the systemic regression of metastatic nodules outside the radiation field after localized irradiation to one or more tumor sites, has attracted significant attention in the last several years, due in part to spectacular breakthroughs in immunotherapies [8,9,10]. Experimental studies conclusively demonstrated that abscopal effects are immune mediated [6,11]. The historic rarity of clinical observations of systemic responses to local radiation is conceivably due to the lack of additional boosts to the immune system [12], as well as infrequent radiological follow-up of untreated metastases. In a retrospective analysis of pre-and post-therapy radiology images of 47 metastatic melanoma cases treated with both ipilimumab and 65 courses of radiation, a shrinking of tumor lesions outside the radiation field was observed in 16 patients, the majority of which had exhibited continued growth after immunotherapy alone [13]. In a recent prospective clinical trial combining focal radiation therapy with immune stimulation in metastatic patients, 11 of 41 patients (26.8%) had an objective abscopal response [9]. The increasing body of literature and evidence that radiation can have robust systemic effects might also change the paradigm of radiation and surgery sequencing for some early-stage localized diseases [14]. Consensus has arisen that the radiation-induced abscopal effect is triggered by stimulation of the adaptive part of the immune system [6,11,15,16], and T cell-mediated responses in particular. Radiation induces cell stress and an immunogenic type of cell death, which activates and primes antigen-presenting cells (APCs) with tumor-associated antigens [17,18]. Activated APCs travel to the tumor draining lymph node where naive CD8+ T lymphocytes can be activated against tumor cells presenting these specific antigens [19]. These newly-activated lymphocytes are distributed systemically by the circulatory system, from which they can also extravasate at unirradiated tumor sites; this is one of the rate-limiting steps for triggering a radiation-induced abscopal effect [7]. Concurrent immunotherapies could augment radiation-induced immune system activation [20], such as immune checkpoint blockade with anti-CTLA-4 antibody to inhibit receptor CTLA-4 [21,22,23] that blocks T cell function. Whilst significant inroads have been made into understanding the radiation-immune system synergy, one of the clinically most relevant questions remains unanswered: What is the best radiation dose and dose fractionation to maximize immune activation? Current radiation protocols have not specifically focused on enhancing immune responses. In fact, fewer, larger doses induce significantly stronger anti-tumor immunity [9,11]. Understanding the complex, non-linear cytotoxic and immunologic consequences of RT, as well as the optimal radiation dose to induce robust anti-tumor immunity is of high biological interest and clinical value. Inroads have been made to compare different radiation schemas for specific cell lines to identify the best of the tested protocols Dewan et al. [11]; yet, limited resources are not permissive of exhaustive preclinical evaluation of all possible dose and dose fractionation protocols [24]. Here, we propose a mathematical framework informed with experimental data that may simulate experimentally untested protocols to help identify radiotherapy protocols optimized for immune activation. For demonstration purposes, but without loss of generality, we present the framework trained on published experimental data of a murine breast cancer model with two co-evolving tumor sites in an in vivo bilateral tumor model (Figure 1).

We develop a mathematical model that simulates radiation response, radiation-induced immune activation, immune checkpoint blockade therapy and the inter-exchange of activated T cells between tumor sites. The proposed model departs from earlier theoretical constructs of predator-prey systems [25] and is first compared to the experimental data from [11]. This data fitting procedure allows us to estimate the model parameters, which are then used to predict responses to dosage not considered in the experimental setting.

## 2. Results

### 2.1. Data Fitting

The optimization procedure provided the same set of estimated model parameters for about 80% of the 1000 randomly-generated initial parameter sets, indicating a robust model structure and that there are only a few local minima. The average volumetric differences between model simulation and experimental data for optimal set of parameters (Table 1) was 16 ± 19 mm^3^ (average relative error of 19.6 ± 16%).

**Figure 2:**
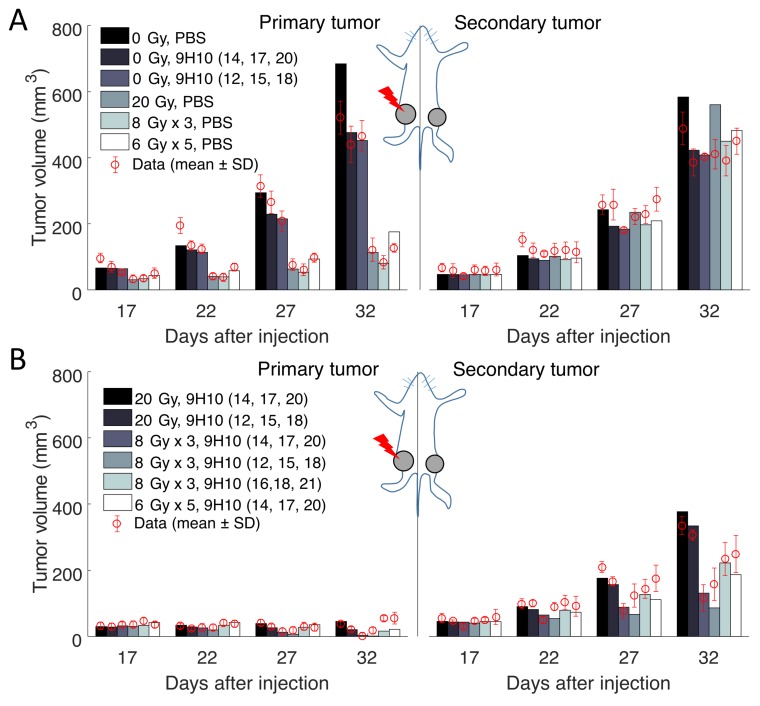
Comparison of the model simulated growth dynamics with experimental data. Solutions to the proposed model (Equations (3)–(10)) were obtained after performing data fitting (estimated parameters are presented in Table 1). (**A**) Experimental and simulated tumor volumes for tumors grown without treatment or after monotherapy. (**B**) Experimental and simulated tumor volumes after combination treatment. Experimental data (red circles) derived from Dewan et al. [11].

**Figure A1:**
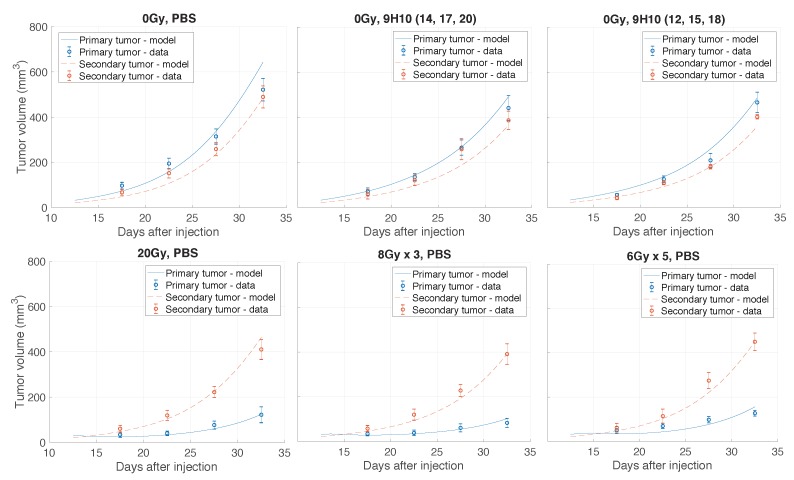
Comparison of model simulated growth dynamics with experimental data. Solutions to the proposed model (lines; Equations (3)–(10)) were obtained after performing data fitting (estimated parameters are presented in Table 1). Shown are experimental and simulated tumor volumes for tumors grown without treatment or after monotherapy. Experimental data (circles) derived from Dewan et al. [11].

**Figure A2:**
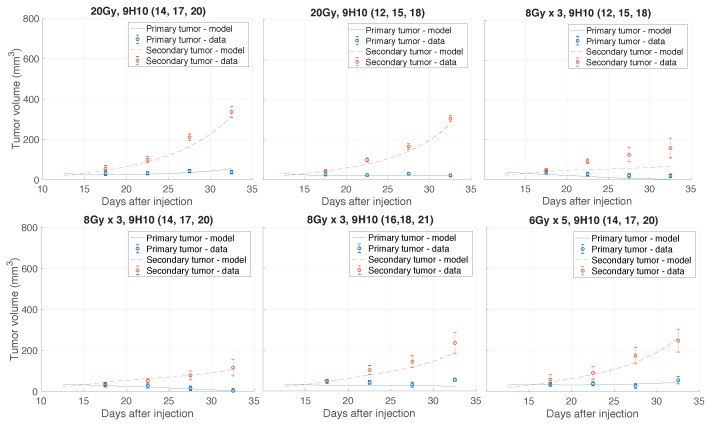
Comparison of model simulated growth dynamics with experimental data. Solutions to the proposed model (lines; Equations (3)–(10)) were obtained after performing data fitting (estimated parameters are presented in Table 1). Shown are experimental and simulated tumor volumes after combination treatment. Experimental data (circles) derived from Dewan et al. [11].

Considering the inherent uncertainty of caliper measurements used in the considered experiment [11], such a low volumetric deviation indicates excellent agreement between the model and analyzed experiment. Figure 2 shows the graphical comparison of measured and simulated tumor volumes for each set of experiments (see Appendix Figure A1 and Figure A2 for a more detailed temporal dynamics visualization).

**Figure 3:**
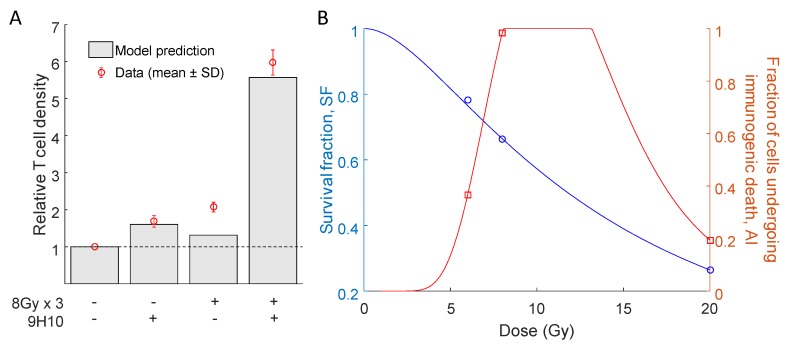
Comparison of relative T cell density and radiation response curves. (**A**) Model simulated and experimentally measured changes in immune cell infiltration at the secondary tumor site after the combination of radiotherapy and 9H10 immunotherapy. (**B**) Proposed interpolation of the model estimated radiation survival fraction ($SF_{D}$) and proportion of cells undergoing immunogenic cell death ($AI_{D}$) for radiation doses *D* = 6, 8 and 20 Gy (see Table 1) using Equations (1) and (2).

Most importantly, the model was able to simulate the synergy of radiation with the immune system correctly. In agreement with the experimental data, only combination therapy yielded a substantial response of the secondary tumor site (Figure 2 right panels). At the same time, there was only a small difference between model predicted and experimentally evaluated changes in immune infiltration of the secondary, non-irradiated tumor (Figure 3A).

Interestingly, model parameters indicated a non-monotonic dependence of the fraction of cells that will undergo immunogenic cell death (AI) on the radiation dose (Table 1, Figure 3B). The model fit to the data suggests that immunogenic cell death was most prevalent after three fractions of radiation with *D* = 8 Gy. With the derived parameter set, the tumor volume radiation survival fraction decreased with increasing radiation dose (Figure 3B).

### 2.2. Predicted Radiation Response

To investigate the response to various radiation fractionation protocols, we needed to interpolate both the values of survival fraction ($SF_{D}$) and the fraction of cells undergoing immunogenic cell death ($AI_{D}$) for radiation doses not tested experimentally. Following Sachs et al. [26], we assumed that the radiation survival fraction can be expressed as:

(1) $$SF_{D}=\exp\left(-\alpha D-\beta G\left(\lambda T\right)D^{2}\right)\;,$$

where $G\left(\lambda T\right)=2\left(\lambda T+\exp\left(-\lambda T\right)-1\right)/{\left(\lambda T\right)}^{2}$, λ is the repair rate, *T* is the delivery time, *D* is the dose and α and β are linear-quadratic model parameters. The above equation was able to fit model-estimated values of $SF_{D}$ for *D* = 6, 8, 20 (see Table 1) for parameter values α = $2.8\times 10^{-8}$, β = 0.0132 and λ = 2.0358 (Figure 3B). It is worth mentioning that the parameters of the radiation response model (1) are conventionally estimated using in vitro clonogenic survival data after 10–14 days. The values reported here refer to in vivo volumetric tumor survival, and thus, the absolute values may not be directly comparable. To interpolate the non-monotonic dependence of the fraction of cells undergoing immunogenic cell death on radiation dose, we used the log-normal distribution without the restriction that the integral over the whole domain needs to be equal to one:

(2) $$AI_{D}=\min\left\{\frac{\gamma }{D}\exp\left(-\frac{{\left(\log\left(D\right)-\mu \right)}^{2}}{\rho }\right),1\right\}\;.$$

Equation (2) is able to fit estimated values of $AI_{D}$ for *D* = 6, 8 and 20 Gy for parameter values γ = 14.173, μ = 2.448 and ρ = 0.232 (Figure 3B).

### 2.3. Optimal Radiation Dose and Dose Fractionation

We simulated the response of both primary and secondary tumors to a single dose irradiation to the primary one and compare final overall tumor burden ($V_{1}$(32$\mathrm{days})+V_{2}\left(32\mathrm{days}\right)$) to simulated responses to radiotherapy divided into multiple fractions with the same total or biologically effective dose (BED). We assumed that radiation is delivered on a daily basis starting from Day 12, and following [27,28], we used the standard BED formula:

BED=nD(1+D/θ).

with θ=20 Gy. In all cases, we simulated concurrent 9H10 immunotherapy using protocols from the experimental setup that was used to calibrate the model.

**Figure 4:**
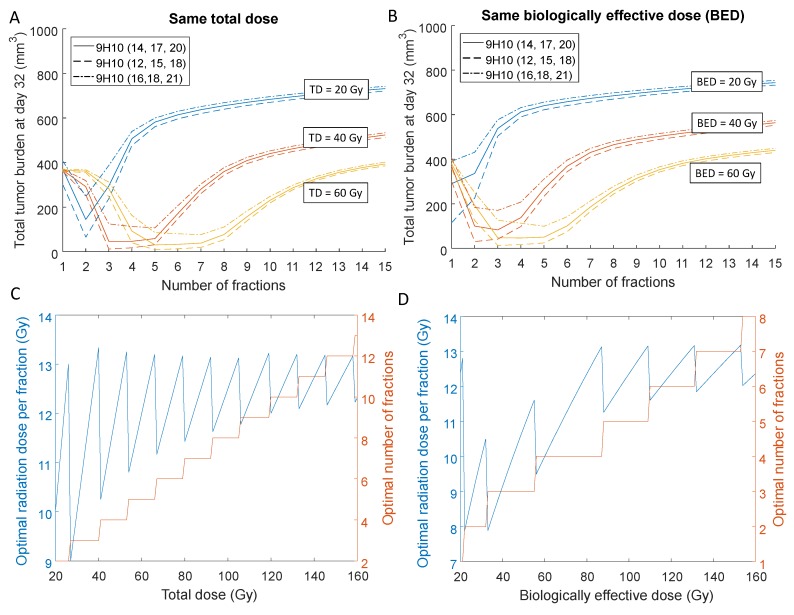
Optimal radiation fractionation and dose per fraction for immune activation. Dependence of the model predicted overall tumor burden at Day 32, i.e., $V_{1}\left(32\mathrm{days}\right)+V_{2}\left(32\mathrm{days}\right)$, for different total (**A**) and biologically effective doses (**B**) for various numbers of fractions. Radiation is delivered daily, and three concurrent doses of 9H10 immunotherapy are applied at different times [11]. (**C**,**D**) Optimal number of radiation fractions and corresponding doses per fraction depending on the prescribed total and biologically effective doses. We assume that 9H10 immunotherapy is applied on Days 12,15 and 18.

The differences in final tumor volumes dependent on radiation fractionation were primarily governed by the response of the secondary tumor as the primary tumor was almost completely eradicated for a total dose of 60 Gy independent of fractionation schedule. Model simulations suggested that the overall tumor response could vary by more than one order of magnitude depending on the radiation protocol. For a total dose of 40 Gy divided into three fractions and immunotherapy administered at Days 12, 15 and 18, the overall tumor burden at Day 32 was 12 mm^3^, compared to 513 mm^3^ if the same total dose was delivered in 15 fractions of 2.67 Gy each (Figure 4A).

Similarly, the model predicts that for 40 Gy BED, the overall tumor burden can be reduced by a factor of almost 20 for the best radiation fractionation (30.5 mm^3^ for 12.36 Gy × 2 compared to 552 mm^3^ for 2.38 Gy × 15; Figure 4B). Moreover, simulations suggest that optimal number of fractions and dose per fraction may allow up to a three-fold reduction of the total dose whilst providing a similar volumetric outcome (53.8 mm^3^ for 7.5 Gy × 8 fractions to a total of 60 Gy total dose vs. 65.3 mm^3^ for 10 Gy × 2 for a total of 20 Gy; Figure 4A), thereby potentially reducing normal tissue toxicity.

Systemic analysis of the optimal number of fractions and dose per fraction, i.e., the number of fractions that leads to the smallest overall tumor burden, for different total and biologically effective doses indicates that there is a certain range of doses per fraction to maximize therapeutic effect. For a clinically relevant total dose (Figure 4C) or biologically effective dose (Figure 4D) of 60 Gy, the model predicted the optimal treatment with 4–5 fractions of 10 Gy–13 Gy each. Model simulations further indicate that timing of immunotherapy within the tested protocols contributed significantly less to overall outcome compared to the radiation fractionation schema. However, the relatively simple model may not be appropriate to investigate timing of immunotherapy thoroughly, as the detailed pharmacokinetics and dynamics of blood absorption from the peritoneal cavity were omitted.

## 3. Discussion

Since the advent of immunotherapy, the synergy of radiation with the immune system has received unprecedented attention. The possibility of rationally inducing systemic anti-tumor immunity with local therapy has the flavor of the long-sought “magic bullet” for cancer cure. While clinical trials for immunotherapy in combination with radiotherapy are plentiful, very little attention is given to a priori optimization of radiation dose and dose fractionation to induce robust anti-tumor immunity. The number of clinically feasible radiation dose and dose fractionations is plentiful and an exhaustive pre-clinical evaluation impossible. Progress in integrated mathematical oncology may render such analyses feasible [24]. Here, we introduced a novel mathematical model calibrated with experimental data to make inroads into deciphering the complexity of radiation and immune system synergy. This framework estimates the optimal number of radiation fractions and radiation dose per fraction to elicit the most pronounced systemic immune-mediated tumor responses for clinically relevant total and biologically effective radiation doses.

Model simulations trained on a specific experimental study suggest that for the chosen cell line in the chosen mouse model, stereotactic radiation may have the highest likelihood of inducing robust anti-tumor immunity, which may contribute to the eradication of the tumor targeted by radiation, as well as tumors and individual cancer cells outside the radiation field. However, to draw conclusions about immune activation, longitudinal blood samples would need to be collected and analyzed for change in circulating immune populations and compared to conventional radiation doses. Standard of care radiation for many localized tumors is delivered in 2-Gy daily fractions for a total of 50–70 Gy [27]. According to model predictions, conventional radiation schemas may not be able to elicit strong immune-mediated tumor responses. It is conceivable that systemic responses would be especially important for patients with lymph node involvement, circulating tumor cells or subclinical or undiagnosed metastatic deposits [14]. This will add an additional layer of complexity to balance the different biological consequences of radiation therapy: induced maximum cell killing whilst sparing immune cells and maximizing subsequent immunity. Recent studies have shown that lymph node irradiation may hinder the maturation and infiltration of immune cells [29], yet lymph nodes are routinely irradiated to eradicate metastatic deposits. While the presented framework may provide a useful tool in preclinical research, caution is warranted before clinical translation. Nevertheless, our work indicates that the results of the recent proof-of-principle trial of 3.5 Gy × 10 fractions with concurrent immunotherapy with more than 25% of patients exhibiting objective systemic responses to focal radiation [9] would be further enhanced if 3–4 stereotactic radiation fractions were applied.

The presented results, however, may be biased by the experimental data used for model calibration. More complex metastatic disease distributions, i.e., combinations of tumors in different organs, need to be evaluated using the proposed framework [7,30]. To increase confidence in the clinical validity of framework predictions, prospective in vivo experiments should evaluate the radiation dose-dependent probability of inducing immunogenic cell death. Nevertheless, the presented work provides the first inroads and quantitative foundations to evaluating radiation fractionation protocols for inducing immune-mediated systemic anti-tumor responses. With the continuously increasing number of clinical trials combining radiation and various forms of immunotherapy, such a framework could become an invaluable tool to evaluate clinical responses, validate the model, help design subsequent clinical protocols and, eventually, support individual patient treatment planning.

## 4. Materials and Methods

### 4.1. Experimental Data

Herein, we use the data reported by Dewan and colleagues in [11], in which TSAmouse breast carcinoma cells were injected subcutaneously into syngeneic mice at two spatially-separated sites (left flank, Day 0, primary tumor; and right flank, Day 2, secondary tumor; Figure 1). Mice received one of four different treatment protocols: Group 1: no treatment; Group 2: irradiation of the primary tumor only with different fractionation protocols: 20 Gy × 1 fraction, 8 Gy × 3 and 6 Gy × 5 (daily doses for each fractionated protocol; radiation delivered at 600 cGy/min); Group 3: three doses of CTLA-4-blocking monoclonal antibody (9H10; immune checkpoint blockade) given at least two days apart, starting on Day 12, 14 or 16; Group 4: combination radiation and immunotherapy (protocol combinations as in Groups 2 and 3). Tumor volumes at both flanks were evaluated every 2–3 days until Day 32. Treatment was initiated when both tumors reached palpable sizes (Day 12; 32 mm^3^ and 21 mm^3^ on average for primary and secondary tumors, respectively). At the end of the experiment (Day 35), secondary tumors from all groups, except for those that received radiation using fractionations other than 3 × 8 Gy, were excised and analyzed by fluorescence microscopy for the presence of CD8+ T cells. The reported mean value at each experimental time point was extracted from [11] for this study.

### 4.2. Mathematical Model of Tumors-Immune System Interaction

We develop a mathematical model that simulates radiation response, radiation-induced immune activation, immune checkpoint blockade therapy and inter-exchange of activated T cells between tumor sites. The proposed model departs from earlier theoretical constructs of predator-prey systems [25]. Based on the experimental setting [11], we consider two tumor sites (i=1,2) at two spatially-separated locations each characterized by time-dependent volume $V_{i}\left(t\right)$. At both sites, we formalize the temporal dynamics of four co-existing and interacting populations: (1) viable cancer cells (volume $C_{i}\left(t\right)$ mm^3^); (2) cancer cells dying in a non-immunogenic manner (volume $D_{i}\left(t\right)$ mm^3^); (3) cancer cells dying in an immunogenic manner (volume $I_{i}\left(t\right)$ mm^3^); and (4) activated tumor-specific cytotoxic T cells (effector cells; density $E_{i}\left(t\right)$ cells/mm^3^). Assuming that immune cells do not contribute significantly to the observed tumor volume, we denote the total measurable volume with:

(3) $$V_{i}\left(t\right)=C_{i}\left(t\right)+D_{i}\left(t\right)+I_{i}\left(t\right).$$

The volume occupied by viable cancer cells is assumed to follow logistic growth with site-independent tumor carrying capacity, *K*, and growth rate, *r*, modulated by the predation of immunocompetent effector cells. Here, carrying capacity and growth rate are assumed to be the same for each tumor site following the experimental setup of cell line-initiated subcutaneous tumors in contralateral flanks of the mouse. The equation governing the growth of each tumor site is:

(4) $$\frac{dC_{i}\left(t\right)}{dt}=rC_{i}\left(t\right)\left(1-\frac{V_{i}\left(t\right)}{K}\right)-aC_{i}\left(t\right)E_{i}\left(t\right).$$

The above equation is analogous to the one considered in [31,32] and in our earlier work [7]. Herein, however, the equation considers population volumes or densities ($C_{i}\left(t\right)$, $V_{i}\left(t\right)$ and $E_{i}\left(t\right)$) instead of individual cell numbers. A detailed description of all parameters with specific values and units can be found in Table 1.

To limit model complexity, we assume that cancer cell death due to competition for resources or immune cell predation is non-immunogenic:

(5) $$\frac{dD_{i}\left(t\right)}{dt}=rC_{i}\left(t\right)\frac{V_{i}\left(t\right)}{K}+aC_{i}\left(t\right)E_{i}\left(t\right)-dD_{i}\left(t\right),$$

in which parameter *d* denotes a fixed clearance rate of dying cells.

After primary tumor ($V_{1}$) irradiation, we introduce an additional volume of dying cells. Radiation-induced cell death is described using the standard approach [27,28]; at each irradiation moment $t_{j}^{+}$, the model system state is instantaneously modified as follows:

(6) $$\begin{array}{ccc} C_{1}\left(t_{j}^{+}\right) & = & C_{1}\left(t_{j}^{-}\right)SF_{D}\;,\\ D_{1}\left(t_{j}^{+}\right) & = & D_{1}\left(t_{j}^{-}\right)+C_{1}\left(t_{j}^{-}\right)\left(1-SF_{D}\right)\left(1-AI_{D}\right)\;,\\ I_{1}\left(t_{j}^{+}\right) & = & I_{1}\left(t_{j}^{-}\right)+C_{1}\left(t_{j}^{-}\right)\left(1-SF_{D}\right)AI_{D}\;, \end{array}$$

where $t_{j}^{-}$ and $t_{j}^{+}$ denote the times immediately before and after irradiation, respectively; $SF_{D}$ denotes the fraction of viable cancer cells surviving radiation with dose *D*; and $AI_{D}$ is the dose-dependent fraction of cancer cells that undergo immunogenic cell death. Consequently, $1-AI_{D}$ denotes the fraction of non-immunogenic cell death events. Here, irradiation is the only source of cells in the compartment $I_{1}\left(t\right)$ from which they are cleared with rate *d*:

(7) $$\frac{dI_{1}\left(t\right)}{dt}=-dI_{1}\left(t\right)\;.$$

We consider APCs that recognize the same tumor-specific antigens at both tumor sites and travel to the tumor-draining lymph node to activate naive T cells. Those activated T cell traffick with the blood system before extravasating at one of the two tumor sites. According to our prior work [7], each trafficking T cell may extravasate at the i-th tumor site with time-dependent probability $p_{i}\left(t\right)$, which in the considered setting can be expressed as:

(8) $$p_{i}\left(t\right)=\frac{V_{i}\left(t\right)}{V_{1}\left(t\right)+V_{2}\left(t\right)}\;.$$

For tumors that are located in different anatomic organs, Equation (8) will include additional terms. However, this could still be simulated with the previously proposed mathematical framework [7]. Without radiation and immunotherapy, effector cells are being recruited proportionally to total volumetric tumor burden $V_{1}\left(t\right)+V_{2}\left(t\right)$. The recruitment term for each tumor site *i* can be expressed as $wp_{i}\left(t\right)\left(V_{1}\left(t\right)+V_{2}\left(t\right)\right)$ where parameter *w* is the overall recruitment rate. Explicit consideration of immunotherapy effects and immunogenic cell death after radiation yields the following equation describing the number of cytotoxic T cells:

(9) $$\frac{dV_{i}\left(t\right)E_{i}\left(t\right)}{dt}=-lE_{i}\left(t\right)V_{i}\left(t\right)+\left(1+u(t)\right)p_{i}\left(t\right)w\sum_{j=1}^{2}\left(1+w_{2}\frac{I_{j}\left(t\right)}{V_{j}\left(t\right)}\right)V_{j}\left(t\right)\;,$$

where the first and second terms are spontaneous exhaustion and recruitment of immune cells, respectively. Function u(t) describes the effect of immunotherapy and under usual pharmacokinetic assumptions [33] can be expressed as:

(10) $$u\left(t\right)=e\int_{0}^{t}c\left(s\right)\exp\left(-clr(t-s)\right)ds\;,$$

where c(s) is drug administration rate at time *s*, clr is the clearance rate of the considered inhibitor and parameter *e* describes the drug impact on the system. Model Equations (3)–(10) have been implemented in MATLAB software and solved using built-in ordinary differential equation solver ode45 with lowered default tolerances (RelToland AbsTolequal to $10^{-8}$) to increase solution accuracy.

### 4.3. Parameter Estimation

The goal of the parameter estimation procedure was to identify model parameters for which the error of: (1) the simulated tumor volumes $V_{1}\left(t\right)$ and $V_{2}\left(t\right)$ compared to experimental volume measurements; and (2) simulated relative change of T cell infiltration in the secondary tumor compared to experimental data,

(11) $$ERR\left(p\right)=\sum_{e\in W_{1}}\sum_{j=1}^{2}\sum_{t_{i}}{\left(\frac{V_{j}\left(t_{i},p\right)-V_{j,data}\left(t_{i}\right)}{V_{j,data}\left(t_{i}\right)}\right)}^{2}+\sum_{e\in W_{2}}{\left(\frac{E_{2}\left(35,p\right)}{E_{2,ctrl}\left(35,p\right)}-R_{data}\right)}^{2}\;,$$

is minimized. The given set of parameters values is denoted by *p*; $W_{1}$ denotes the set of all experiments; $W_{2}$ denotes the set of experiments in which immune infiltration in the secondary tumor was evaluated at Day 35; $t_{i}$ denotes one of the recorded measurement times; ctrl denotes control experiment (no radiation and immunotherapy); and $R_{data}$ denotes the measured change in immune infiltration relative to the control experiment.

Although 9H10 immunotherapy was injected intraperitoneally in the considered experiment [11], we assume for simplicity that $c\left(s\right)=D(\delta \left(s-{\bar{t}}_{1}\right)+\delta \left(s-{\bar{t}}_{2}\right)+...)$, where δ is the Dirac delta function and $t_{i}$ is injection day (we take e=eD in Equation (10)). This assumption may in general lead to a decrease of fit quality and introduces inherent bias towards applying immunotherapy at the same time as radiation, but at the same time, we do not need to estimate additional parameters related to drug release from the intraperitoneal cavity.

For parameter estimation, 124 data points were used to calibrate the values of 13 parameters simultaneously. Only values of two parameters were kept fixed during the minimization process: (1) tumor growth rate *r*, which was calculated directly from the initial growth data of untreated control experiments; (2) T cell exhaustion rate *l*, which was taken directly from [34]. For numerical error minimization, we used the deterministic derivative-based trust region method [35] implemented in the lsqnonlin function from MATLAB’s Optimization Toolbox. In order to avoid local minima, we generated 1000 random initial parameter values for the optimization procedure.

## Figures and Tables

**Table 1:** Model (Equations (3)–(10)) parameters and derived values.

Parameter	Description	Unit	Value
*r*	Viable cancer cells volume doubling time	1/day	0.195
*K*	Tumor carrying capacity	mm^3^	1423.1
*a*	CTLs’ killing rate	mm^3^/(cell·day)	0.0177
*d*	Clearance rate of dying cells	1/day	0.264
$SF_{D}$	Fraction of viable cancer cells that survive		
	after radiation dose		
	D=20 Gy	-	0.265
	D=8 Gy	-	0.664
	D=6 Gy	-	0.783
$AI_{D}$	Fraction of cells that will undergo		
	immunogenic cell death after		
	radiation dose		
	D=20 Gy	-	0.194
	D=8 Gy	-	0.984
	D=6 Gy	-	0.367
*l*	Decay rate of effector cells	1/day	0.03
*w*	Baseline T cell recruitment rate	cell/ (mm^3^·day)	0.135
$w_{2}$	Fold change in the baseline T cell recruitment		
	rate due to immunogenic cell death	-	15.37
*e*	Initial fold change in recruitment		
	of cytotoxic T cells caused by the administered		
	dose of 9H10 immunotherapy	-	8.495
clr	9H10 immunotherapy clearance rate	1/day	0.967

## Abbreviations

The following abbreviations are used in this manuscript:

RT	Radiotherapy
APC	Antigen-presenting cell
CTL	Cytotoxic T lymphocyte
IR	Irradiation
BED	Biologically effective dose
